# Chronic intermittent hypoxia increases airway hyperresponsiveness during house dust mites exposures in rats

**DOI:** 10.1186/s12931-023-02493-4

**Published:** 2023-07-19

**Authors:** Mihaela Teodorescu, Ruolin Song, Jacqueline A. Brinkman, Ronald L. Sorkness

**Affiliations:** 1 Department of Medicine, School of Medicine and Public Health, University of Wisconsin— Madison, Madison, WI USA; 2 William S. Middleton Memorial VA Medical Center, Madison, WI USA; 3 Division of Allergy, Pulmonary and Critical Care Medicine, University of Wisconsin School of Medicine and Public Health, William S. Middleton Memorial Veterans’ Hospital, 2500 Overlook Terrace, D2212, Madison, WI 53705 USA; 4 School of Pharmacy, University of Wisconsin—Madison, Madison, WI USA

**Keywords:** Asthma, Sleep apnea, Obstructive, Intermittent hypoxia, Allergic airway inflammation, Lower airway, Airway hyperresponsiveness, Methacholine chloride/pharmacology, Rats

## Abstract

**Introduction:**

Accumulating clinical evidence links Obstructive Sleep Apnea (OSA) with worse outcomes of asthma, but impact on airway function remains sparsely studied. We tested effects of Chronic Intermittent Hypoxia (CIH) – a hallmark of OSA – on airway hyperresponsiveness (AHR), in a rat model of chronic allergen-induced inflammation.

**Methods:**

Brown Norway rats were exposed to six weeks of CIH or normoxia (NORM) concurrent with weekly house dust mites (HDM) or saline (SAL) challenges. At endpoint, we assessed responses to seven Methacholine (Mch) doses (0, 4, 8, 16, 32, 64, 128 mg/mL) on a FlexiVent system (Scireq). Maximal (or plateau) responses (reactivity) for total respiratory system Resistance (R_rs_) and Elastance (E_rs_), Newtonian airway resistance (R_N,_ a measure of central airways function) and tissue damping (G, a measure of distal airways function) were plotted.

**Results:**

HDM/CIH–treated animals demonstrated the highest reactivity to Mch in R_rs_ and E_rs_ compared to all other groups (HDM/NORM, SAL/CIH and SAL/NORM *p* < 0.05 for all comparisons, for doses 5–7 for R_rs_, and for doses 4–7 for E_rs_). The enhanced R_rs_ response was due to an increase in G (doses 4–7, *p* < 0.05 for comparisons to all other groups), whereas R_N_ was not affected by CIH.

**Conclusions:**

In rats chronically challenged with HDM, concurrent CIH exposure induces AHR primarily in the distal airways, which affects the respiratory system frequency-dependent elastic properties.

## Introduction

Asthma is a common chronic pulmonary disease, in the US, for example, affecting ~ 8% and 7% of adults and children, respectively [1]. Asthma is associated with significant morbidity and mortality [1] with the final, fatal attacks occurring predominantly at night [2, 3]. Obstructive Sleep Apnea (OSA) is ~ 2.6 fold more common in patients with asthma [4] where it is associated with poor disease control (reviewed in [5]) and increased risk for exacerbations [6–8]. Conversely, OSA treatment with Continuous Positive Airway Pressure (CPAP) improves daytime and nighttime asthma symptoms, rescue bronchodilator use, exacerbations, disease-specific quality of life, and A.M. and P.M. peak expiratory flow rates [7, 9–14].

Pathophysiologically, underlying asthma is lower airway inflammation, hyperresponsiveness and remodeling, leading to airflow obstruction and clinical expression of symptoms [15]. Airway hyperresponsiveness (AHR) can result from narrowing of the central airways, small airways, or a combination of both, as well as plugging or closure of small airways and alveoli [16, 17]. It has a heterogenous substrate consisting of inflammation on the background of smooth muscle contraction/hypertrophy and tissue remodeling [16, 18]. OSA is characterized by recurrent episodes of transient closure in the upper airway, associated with interruption in the airflow, leading to alteration in gas exchange, such as Chronic Intermittent Hypoxia (CIH), increased work of breathing and sleep fragmentation, which ultimately terminate the event and re-establish the upper airway patency [19]. How these pathophysiologic hallmarks of OSA modulate asthma’s features has been only sparsely studied. We previously reported that exposure to CIH during allergic lower airway inflammation in rats exacerbated the airway dysfunction through a departure from traditional Th2 eosinophilic to a Th1 pattern of inflammation, associated with heterogenous lung tissue remodeling that consisted of larger airway wall fibrosis and small airway basement membrane thinning, and “emphysema-like” formations in the lung periphery [20].

It remains unknown how CIH of OSA impacts AHR associated with asthma. Herein, we tested the effects of CIH on lower airway responses to methacholine and their compartmental distribution, during chronic allergen-induced airway inflammation in rats. We hypothesized that CIH increases AHR during allergic exposure along the lower airway compartments. Preliminary results of this study were presented in abstract form [21].

## Methods

### Animals

Male, 7–11-week-old Brown Norway (Strain 091, Charles River Laboratories, Wilmington, MA) rats (n = 10–12/ group) were used for this study. They were housed two to four per cage and provided with standard rat chow and drinking water ad libitum.

### Experimental protocol

As we previously reported in this model [22], on Day 1 and 3, all animals were sensitized to House Dust Mites (HDM) extract (from *Dermatophagoides pteronyssinus*, Stallergenes Greer Laboratories, Lenoir, NC), 5 mg total protein in 250mL sterile saline by intratracheal instillation. Following recovery on day 1, rats began 6-week exposures to normoxia (NORM) or CIH (10% FiO_2,_ 30 cycles/h, 10 h/day during the light cycle), produced as we previously reported [20]. Commencing 6 days after first sensitization (day 7), weekly HDM (10 µg total protein in 250mL sterile saline) or sterile saline (SAL, 250mL) challenges were given intratracheally. Thus, four experimental groups were studied: HDM/CIH, HDM/NORM, SAL/CIH and SAL/NORM. HDM allergen was used given its ubiquitous presence in the environment and relevance to human exposures.

### Measurements and outcomes

Pulmonary function with methacholine (Mch) challenge testing (MCT) and blood collection for HDM-specific IgE titers were performed per our established methods [22, 23]. In brief, two days after last challenge (day 44), rats were anesthetized (urethane), tracheotomized and fitted with a metal endotracheal cannula (Harvard Apparatus) and placed on a mechanical ventilator (FlexiVent FX4 system, Scireq, Montreal, QC, Canada) equipped with an in-line drug nebulizer. The animals were ventilated with a tidal volume of 10 mL/kg body weight, 90 breaths/min and 3 cmH_2_O positive end-expiratory pressure. They were paralyzed with succinylcholine to eliminate any breathing effort artifact during pulmonary function testing (PFTs). At first, a baseline scan was obtained, followed by MCT with incremental doses of Mch  (4, 8, 16, 32, 64 and 128 mg/mL) diluted in SAL and nebulized over 10 s at 50% duty cycle. After the baseline scan and prior to nebulizing each Mch dose, animals were suctioned with a polyethylene catheter (PE-50, internal/external diameters 0.53 / 0.97 mm, length 12 cm; Becton-Dickinson).

For outcomes, the FlexiVent system allows assessing pulmonary airway function along with partitioning the contribution of its central and distal compartments. Specifically, parameters obtained included overall respiratory system resistance (R_rs_) and quasi-static elastance (E_rs_), Newtonian resistance (R_N_, a measure of central airways function) and tissue damping (G, reflecting energy dissipation in the peripheral tissue due to internal friction, and an index of distal airway function). For each parameter, measurements were repeated 12 times and the maximum value among all valid measurements was taken. We assessed AHR as maximal (or plateau) response (Mch reactivity) by plotting the dose response curves of maximum values for each parameter, across all Mch doses (0-128 mg/mL) tested.

Following PFTs, animals were euthanized and blood was sampled from the portal vein and kept at room temperature for ~ 30 min, for clotting to occur. Samples were subsequently spun down at 2000 g and 4 °C, for 10 min. The supernatant (serum) fraction was harvested for measurement of anti(α)-HDM-specific IgE levels using sandwich ELISA, as we previously reported [22].

### Statistical analysis

Data are summarized as means ± standard errors of the mean (SEM). We used 2-way ANOVA (repeated measures [RM] design where appropriate) with Holm-Sidak *post hoc* tests to compare individual group data and multiple comparison-adjusted *p*-values being reported. *P*-values less than 0.05 were considered statistically significant and those 0.05–0.10 were considered trends. Statistical analyses and graphing were performed using Prism 9.0 software (GraphPad, San Diego, CA, USA).

## Results

### CIH did not substantially affect the HDM-induced alterations in baseline pulmonary function

**Fig. 1:**
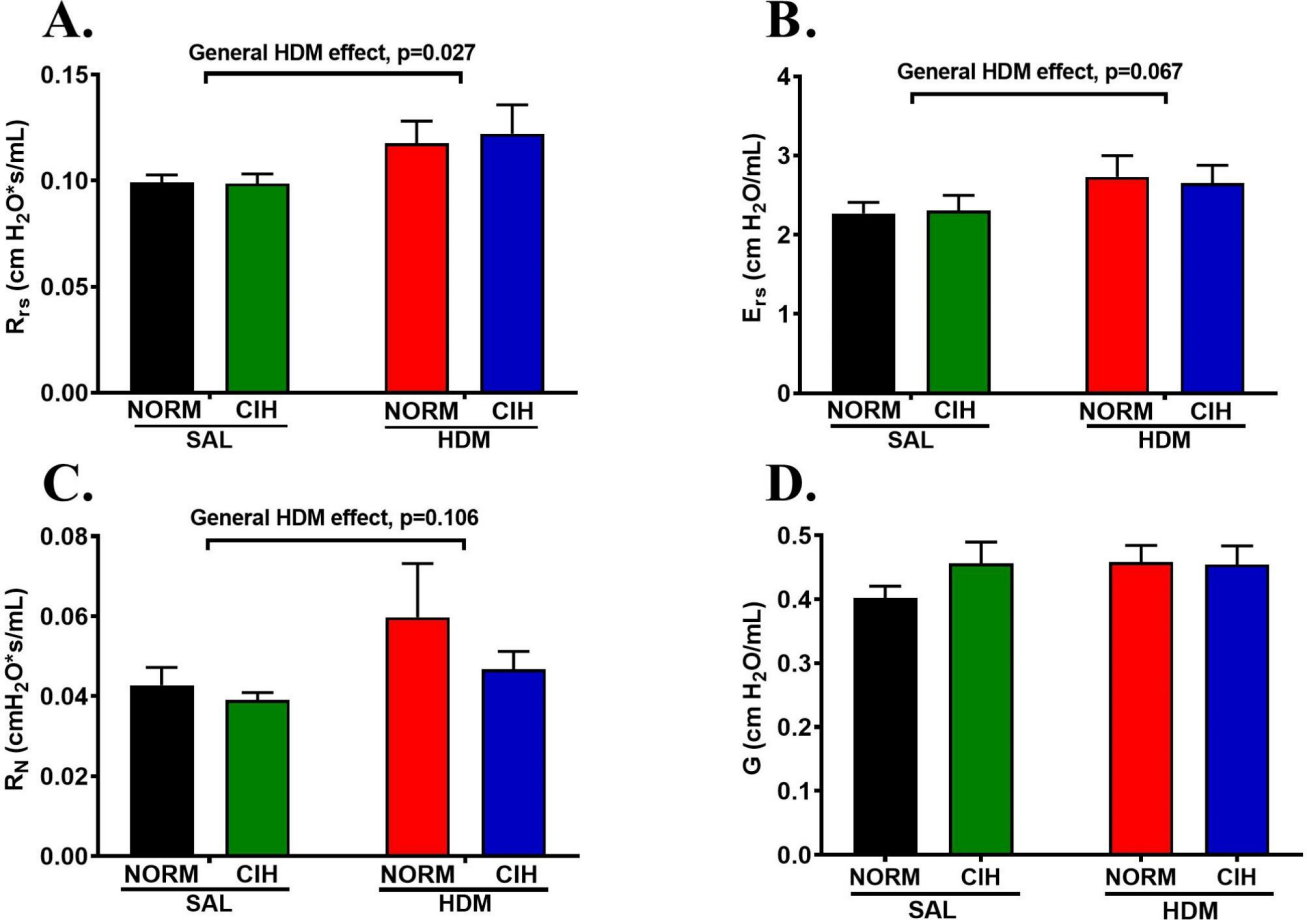
CIH exposure did not substantially alter baseline pulmonary function. Baseline lung physiology was tested on day 44, two days after last HDM or SAL challenge, using the FlexiVent. **(A)** total respiratory system resistance (R_rs_); **(B)** total respiratory system elastance (E_rs_); **(C)** Newtonian resistance (R_N_, index of central airways resistance), and; **(D)** tissue damping (G, index of distal, peripheral airway resistance). N = 10–12 rats/group; *Bars*: mean ± SEM; *Accolades*: general or group effect of HDM (2-way ANOVA and Holm-Sidak post-hoc tests for group comparisons)

In this chronic model, HDM exposure induced modest detrimental effects on baseline pulmonary function, consisting of an increase in total respiratory system resistance (R_rs_, general effect of HDM, *p* = 0.027) (Fig. 1A), which tended to adversely affect the system elastic properties and elevate respiratory system elastance (E_rs_, general effect of HDM, *p =* 0.067) (Fig. 1B). Among airway compartments, HDM challenges tended to increase central airway resistance (R_N_, general effect of HDM, *p =* 0.106) (Fig. 1C) and had no effect on tissue damping (G), the index of distal airway function (Fig. 1D). CIH did not alter any of these HDM-induced effects on the baseline pulmonary function, in either SAL or HDM challenged rats (Fig. 1).

### CIH increased the AHR to Mch

**Fig. 2:**
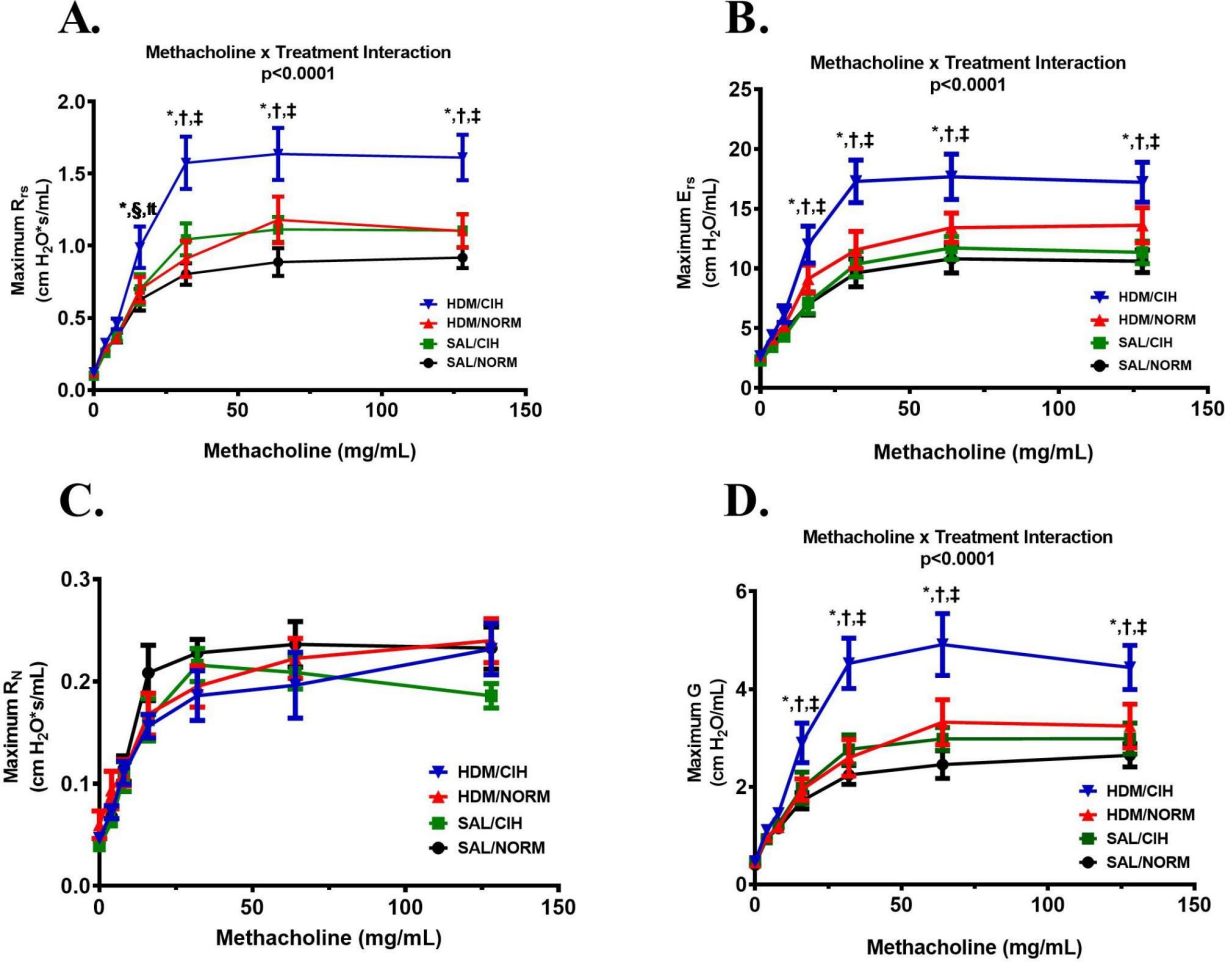
CIH increased Methacholine reactivity expressed primarily in the distal airway. The maximal responses (reactivity) for respiratory system resistance (R_rs_) **(A)** and elastance (E_rs_) **(B)**, Newtonian resistance (R_N_) **(C)** and tissue damping (G) **(D)** recorded across Methacholine concentrations of 0, 4, 8, 16, 32, 64 and 128 mg/mL. HDM/CIH group significantly different vs.: ^*^SAL/NORM, ^†^SAL/CIH and ^‡^HDM/NORM (all *p* < 0.05); HDM/CIH group trends relative to ^§^SAL/CIH and ^#^HDM/NORM (*p =* 0.056 for each comparison); (2-way [RM] ANOVA and Holm-Sidak post-hoc tests for group comparisons). Data presented as mean ± SEM. N = 10–12 rats/group

As shown in Fig. 2A, R_rs_ increased with higher Mch doses, across all groups (*p* < 0.0001 for each group). A significant Mch x treatment interaction (*p* < 0.0001) was also seen, explained by the significant differences between HDM/CIH–challenged rats relative to all other groups. Specifically, these animals demonstrated significantly steeper early responses and achieved higher plateaus, indicative of increased reactivity. This augmentation of system resistance was accompanied by an increase in the system elastic properties (elastic recoil) (Fig. 2B). With higher Mch doses, E_rs_ increased across all groups (*p* < 0.0001 for each group). A significant Mch x treatment interaction (*p* < 0.0001) was also seen, likewise due to enhanced responses in the HDM/CIH rats compared to the other groups. These system responses were due to an increase in maximum G (Fig. 2D), which showed similar dose-response curves kinetics as the R_rs_, with an overall Mch effect across all groups (*p* < 0.0001 for each group) and a Mch x treatment interaction *p* < 0.0001 related to HDM/CIH differences from all other groups at most Mch doses (4 to 7). Although a similar increase in R_N_ with Mch was noted across all groups (p < 0.0001 for each group), no significant group differences were noted in this parameter (Mch x treatment interaction *p* = 0.435) (Fig. 2C).

### CIH upregulated the allergic response to HDM

**Fig. 3:**
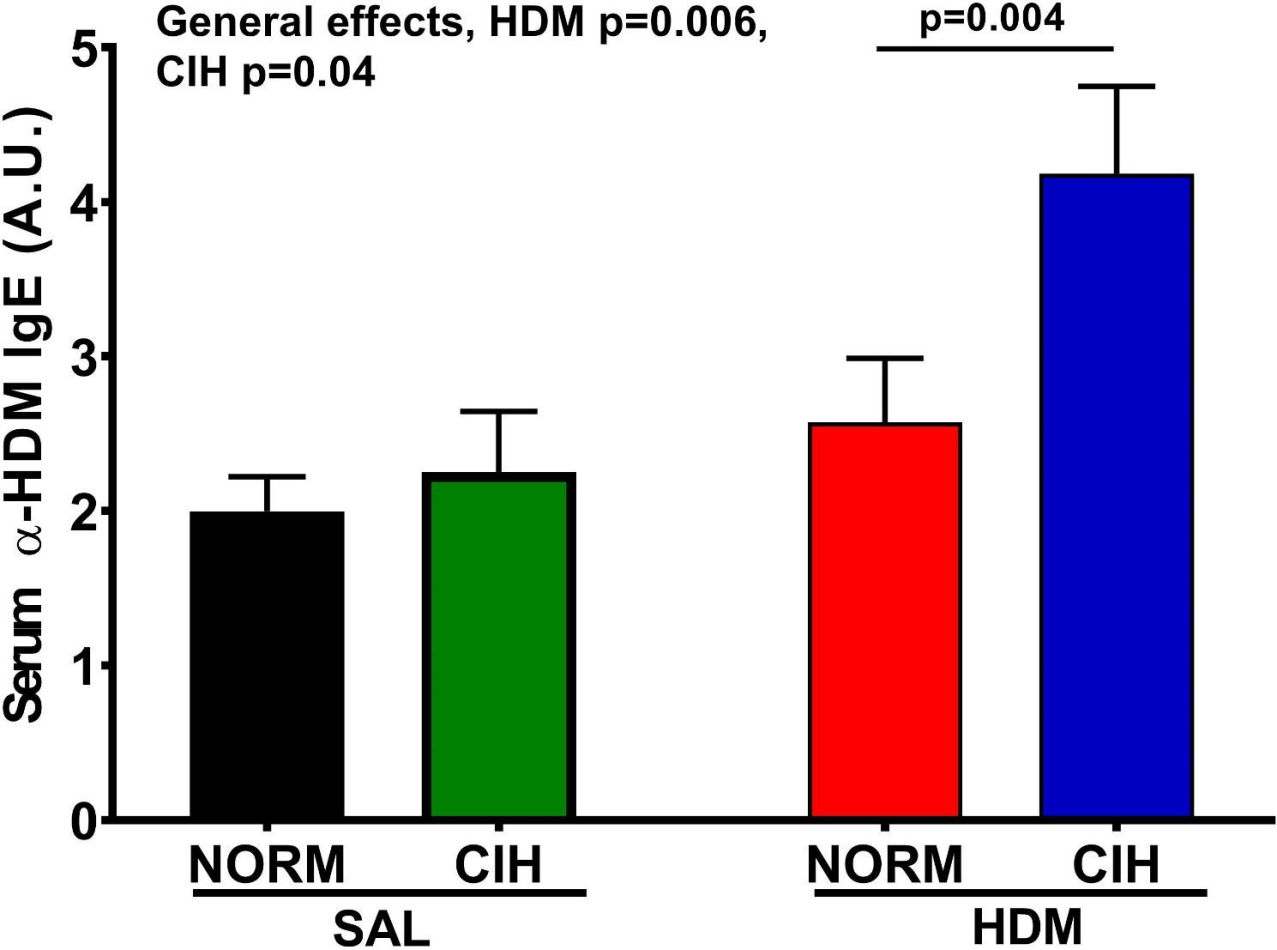
Serum HDM-specific IgE level was significantly increased in HDM-challenged animals. At the endpoint (day 44), blood was collected and HDM-IgE level assayed in serum by ELISA. An overall effect of HDM was found, of greatest magnitude among CIH-exposed animals. General effects of CIH and HDM *p*-values shown; *Accolades*: group effect of CIH *p*-values shown (2-way ANOVA and Holm-Sidak post-hoc tests for group comparisons). Data presented as mean ± SEM; N = 8–10 rats/ group

HDM compared to SAL challenges significantly increased serum specific α-HDM IgE levels (Fig. 3, general HDM effect, *p =* 0.006). A general CIH effect was also present (*p* = 0.04) with the largest IgE levels noted in the HDM/CIH treated animals (62% relative increase vs. HDM/NORM, *p* = 0.004) (Fig. 3).

## Discussion

AHR is a core pathophysiologic feature of asthma, and a predictor of exacerbations and response to therapies [18, 24]. Because OSA relates to poor clinical outcomes in asthma (reviewed in [5]) including risk for exacerbations [6–8], and CIH—its hallmark feature—exacerbates baseline allergic airway dysfunction [20], herein, we report on the first study testing the CIH effects on AHR during a 6-week exposure to a naturally occurring environmental aeroallergen. We found that in chronically HDM-challenged rat, CIH: (1) did not substantially modulate HDM-induced baseline airway dysfunction (Fig. 1), but; (2) increased AHR, to the largest degree noted in HDM-challenged rats (Fig. 2A), which was; (3) expressed primarily in the distal airways (Fig. 2D), and; (4) detrimentally affected the elastic properties of the respiratory system (Fig. 2B). These data emphasize the potential of CIH of OSA to modulate airway behavior in response to aeroallergens, increase its instability and propensity to asthma exacerbations.

A partly analogous pattern of pulmonary physiologic findings has been reported in patients with asthma and OSA (reviewed in [5]). Specifically, a lack of association of OSA with airway dysfunction on daytime spirometry was reported in one correlative study [25], as well as no impact of CPAP treatment for OSA on spirometric measures [7, 10, 13]. With respect to OSA’s effects on airway reactivity, reports are mixed, with a significant reduction with CPAP therapy in the proportion of patients with a positive bronchodilator response [7] whilst no change in the Mch PC_20_ [13]. In this later study, one possible explanation for the negative results stems from the subjects’ selection focused on those with no variability (more than 2 dilutions) in Mch PC_20_ on three serial baseline tests obtained 2–3 days apart. Inherent to asthma is its variability in the degree of airway dysfunction, which is a marker of unstable disease [15]. Therefore, presumably, subjects who were most reactive to Mch were also most likely to experience benefit from CPAP and excluded from the study. Thus, the issue of OSA’s effects on AHR in asthma remains unsettled and needs to be thoroughly studied in well-defined patient populations.

The augmentation of AHR expression by CIH predominantly in the distal airway of HDM-challenged rats sheds some initial mechanistic insight, pointing towards contribution from exaggerated closure of smaller airways, possibly related to remodeling. We have previously reported structural “remodeling” along the airway, in Ovalbumin (OVA)-challenged Brown Norway rats exposed to even a shorter (4-week) duration of CIH, of the same daily intensity [20]. Although the muscular layer was not assessed in that study, notably, in the distal airway (< 500 μm bronchial basement membrane [BBM] perimeter), we observed thinning of the BBM accompanied by upregulated alveolar size, i.e., “emphysema-like” formations in the lung periphery [20]. Likewise, Taille et al., reported thinner BBM on endobronchial biopsy specimens from patients with severe asthma and OSA vs. those without OSA, and a negative correlation of BBM thickness with OSA severity measured by apnea-hypopnea index (AHI) [26]. Collectively these data lead us to posit that in the current model, such structural airway and parenchymal changes in the face of a triggering exposure could reduce the airway wall stiffness and airway-parenchymal tethering, enabling premature distal airway closure to occur during the bronchoconstrictive response [16]. Further studies in this model are necessary to evaluate the BBM properties along the airway compartments, as well as other potential contributors to AHR, such as the muscular layer (mass, fiber contractile function), balance (density, activity, etc.) of broncho-active receptors (muscarinic, adrenergic) in the airway compartments and the sensitivity of vagal a/efferents to the muscle [27].

Our study is not devoid of limitations. One relates to lack of assessment of airway inflammation—another key feature of the pathologic airway process—intricated with the expression of AHR [18, 28]. The heterogeneity of airway inflammation in asthma with a role of non-Th2 pathways in the disease severity and fatality is increasingly recognized [29]. The observations from clinical studies that AHR occurs in the absence of sputum eosinophilia [24, 30] and that therapies targeting Th2 inflammation do not eliminate AHR despite effectively suppressing airway eosinophils to levels comparable to controls [28], give rise to the question that other inflammatory cells also play a role in AHR. Our 4-week OVA/CIH model uncovered a shift from Th2 to Th1-type of inflammation in the bronchoalveolar lavage (BAL) fluid, with a predominance of monocytes and macrophages that evoked more of a pro-fibrotic/remodeling phenotype. Moreover, with a 2-week CIH exposure, albeit in the absence of allergy, Low et al., also showed a Th1 type, neutrophilic predominant cellularity and elevated IL-6 levels in the BAL [31]. Together, these studies indicate the potential of CIH to modulate the airway inflammatory process towards non-Th2 pathways with an evolving cellular type predominance over time. The time dependence and how this type of inflammation links with AHR along the airway compartments in this model need to be tested. Secondly, the modest overall HDM vs. SAL effects on basal airway dysfunction, AHR and IgE responses indicate that our once-a-week HDM challenge frequency may have been too low, but still, CIH significantly impacted some of the HDM responses (Figs. 2 and 3). Although data in rats are lacking, a recent study in mice has shown that a 3-5x/week HDM challenge frequency is necessary to elicit aforementioned responses in protocols of 4–8 weeks duration, the shorter the protocol the more numerous challenges required [32]. While further dosing optimization is needed in this model, given the observed impact of CIH, as a cautionary note, one must consider the potential for saturation achieved by more intense HDM exposures leaving little room for the CIH effects to be expressed.

*In conclusion*, CIH concurrent with HDM exposure in rats induced AHR arising predominantly in the peripheral airway. These findings suggest airway instability and could relate with increased risk for exacerbations in patients with asthma. Additionally, they suggest a role of CIH of OSA in the origins of peripheral airway dysfunction in asthma, i.e. “silent zone”. Our observations call for further studies to understand their mechanistic basis and thoroughly assess OSA’s role in the origins of “silent zone”, aimed at informing targeted therapies for patients living with asthma/OSA overlap.

## Data Availability

The datasets used and/or analyzed during the current study are available on reasonable request. Requests for access can be sent to: Lynn Tarpey, Privacy/FOIA Officer, William S. Middleton Memorial Veterans Hospital, Madison, WI. Phone: 608-256-1901 × 11699, Email: MadisonFOIA@va.gov.

## Abbreviations

AHI: Apnea Hypopnea Index
BBM: Bronchial Basement Membrane
CIH: Chronic Intermittent Hypoxia exposure
CPAP: Continuous Positive Airway Pressure
FEV1: Forced Expiratory Volume in first second of the Forced Vital Capacity
FVC: Forced Vital Capacity
HDM: House Dust Mites allergen
NORM: Normoxia exposure
PC_20_: Provocative Concentration of methacholine causing a 20% fall in FEV_1_ from baseline
OSA: Obstructive Sleep Apnea
PFTs: Pulmonary Function Tests
SAL: Saline
